# Proteomics reveals unique identities of human TGF-β-induced and thymus-derived CD4^+^ regulatory T cells

**DOI:** 10.1038/s41598-022-23515-z

**Published:** 2022-11-24

**Authors:** Mark Mensink, Ellen Schrama, Eloy Cuadrado, Derk Amsen, Sander de Kivit, Jannie Borst

**Affiliations:** 1 Department of Immunology and Oncode Institute, Leiden University Medical Center, Leiden, The Netherlands; 2 Department of Hematopoiesis, Sanquin Research and Landsteiner Laboratory, Amsterdam UMC, University of Amsterdam, Amsterdam, The Netherlands

**Keywords:** Regulatory T cells, Proteome informatics

## Abstract

The CD4^+^ regulatory T (Treg) cell lineage, defined by FOXP3 expression, comprises thymus-derived (t)Treg cells and peripherally induced (p)Treg cells. As a model for Treg cells, studies employ TGF-β-induced (i)Treg cells generated from CD4^+^ conventional T (Tconv) cells in vitro. Here, we describe how human iTreg cells relate to human blood-derived tTreg and Tconv cells according to proteomic analysis. Each of these cell populations had a unique protein expression pattern. iTreg cells had very limited overlap in protein expression with tTreg cells, regardless of cell activation status and instead shared signaling and metabolic proteins with Tconv cells. tTreg cells had a uniquely modest response to CD3/CD28-mediated stimulation. As a benchmark, we used a previously defined proteomic signature that discerns ex vivo naïve and effector Treg cells from Tconv cells and includes conserved Treg cell properties. iTreg cells largely lacked this Treg cell core signature and highly expressed e.g. STAT4 and NFATC2, which may contribute to inflammatory responses. We also used a proteomic signature that distinguishes ex vivo effector Treg cells from Tconv cells and naïve Treg cells. iTreg cells contained part of this effector Treg cell signature, suggesting acquisition of pTreg cell features. In conclusion, iTreg cells are distinct from tTreg cells and share limited features with ex vivo Treg cells at the proteomic level.

## Introduction

Immune responses depend on a balance between the opposing activities of conventional (Tconv) and regulatory T (Treg) cells^1^. While Tconv cells are key mediators of adaptive immunity, Treg cells are specialized in maintaining immune tolerance and controlling inflammation^1^. Based on their origin, Treg cells can be classified into two main groups. Thymus-derived (t)Treg cells are primarily directed at self-antigens and stably express the Treg cell master transcription factor FOXP3, which is indispensable for their identity and suppressive function^2–6^. In addition, they express the transcription factor Helios (IKZF2), which is consequently used as a marker of stable Treg cells^7,8^. Peripherally induced (p)Treg cells are converted from Tconv cells in response to foreign antigens^9^ and reportedly have less stable FOXP3 expression^10–13^, in particular recently after their development^14^. Stable expression of FOXP3 is established epigenetically by demethylation of several regions within the *FOXP3* gene locus, including the so-called Treg-specific demethylated region (TSDR) in the CNS2 enhancer^5,15^.

Based on expression of CD45RA, human Treg cells from peripheral blood can be divided into naïve (CD45RA^+^) and effector (CD45RA^–^) Treg cells^16^. Since conversion of Tconv cells into pTreg cells requires their activation, it stands to reason that the naïve Treg cell population is mainly composed of tTreg cells^17,18^. Such naïve Treg cells can differentiate into effector Treg cells in response to inflammatory signals^19^. Therefore, the effector Treg cell population arguably consists of a mixture of pTreg cells and activated tTreg cells. Amongst human effector Treg cells, it is currently not possible to definitively discriminate tTreg and pTreg cells for a lack of cell surface markers^20^.

Naïve Treg cells occur in low abundance in the blood^21,22^ and effector Treg cells from the blood are not amenable to expansion in vitro^16,23^. For these and other reasons, studies that investigate Treg cell differentiation, function or metabolism in vitro often employ TGF-β-induced (i)Treg cells that are generated from Tconv cells^24–27^. Such iTreg cells resemble Treg cells by their expression of FOXP3 and their suppressive capacity^28,29^. However, in terms of FOXP3 expression and suppressive function, iTreg cells are less stable than tTreg cells. For example, adoptively transferred iTreg cells in mice lost Foxp3 expression and failed to prevent graft-versus-host disease, in contrast to tTreg cells^26^. Similarly, others showed that iTreg cells had reduced Foxp3 expression and suppressive capacity after removal of TGF-β in vitro and that the majority of iTreg cells rapidly lost Foxp3 expression following adoptive transfer in wild-type mice^30^.

Despite the acquisition of Treg cell-like features, it is unclear whether iTreg cells represent an equivalent of ex vivo Treg cells. We recently described that ex vivo naïve and effector Treg cells from human peripheral blood have a common proteomic signature that sets them apart from other CD4^+^ T cell subsets^22^. This Treg cell core signature reflects unique Treg cell properties associated with signal transduction, transcriptional regulation and cell metabolism. In addition, we published a proteomic signature that is unique to ex vivo effector Treg cells as opposed to Tconv cells and naïve Treg cells^22^. Using these proteomic signatures as benchmarks, we aimed to determine to what extent in vitro-generated iTreg cells resemble ex vivo Treg cells. The recently identified cell surface molecule GPA33, which marks naïve Treg cells in human blood, enabled us to include in our comparative study highly pure tTreg cells, without using the mTOR inhibitor rapamycin^21,22^. In brief, our proteomic analysis shows that iTreg cells have minimal features in common with ex vivo Treg cells. We report large differences between human iTreg, tTreg and Tconv cells in terms of protein expression and biological processes, providing a proteomic resource for further research into human Treg cells.

## Results

### Validation of Tconv, tTreg and iTreg cell populations used in this study

**Figure 1:**
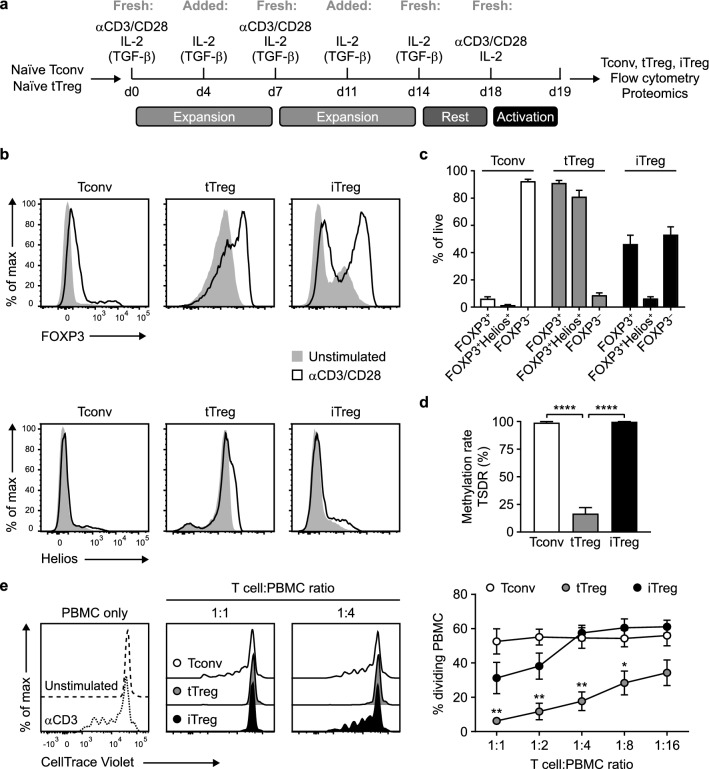
Characterization of Tconv, tTreg and iTreg cell populations. **(a)** Schematic overview of expansion protocols for Tconv, tTreg and iTreg cells. TGF-β was used to generate iTreg cells. Prior to analysis, expanded cells were restimulated for 24 h or not, as indicated in figure legends. **(b)** Flow cytometric analysis of FOXP3 and Helios (IKZF2) protein levels in Tconv, tTreg and iTreg cells at 24 h after restimulation with anti-CD3/CD28 mAbs or unstimulated control (representative of *n* = 7). **(c)** Quantification of the frequency of FOXP3^+^, FOXP3^+^Helios^+^ and FOXP3^−^ cells in the resting Tconv, tTreg and iTreg cell populations after expansion (*n* = 7). **(d)** Analysis of the methylation status of the TSDR in the *FOXP3* locus in Tconv, tTreg and iTreg cells. One-way ANOVA with Tukey’s post hoc test was used for statistical analysis (*n* = 6–7). **(e)** Assessment of the suppressive capacity of Tconv, tTreg and iTreg cells that were cocultured for 4 days with CellTrace Violet-labeled responder peripheral blood mononuclear cells (PBMCs) in the presence of agonistic mAb to CD3. Representative flow cytometry plots are shown in the left panel. Quantification showing the percentage of dividing PBMCs is displayed in the right panel. One-way ANOVA with Bonferroni’s post hoc test was used for statistical analysis (*n* = 4). **(c–e)** Data are presented as mean ± SEM. Sample size (*n*) represents cells from individual donors, analyzed in independent experiments. *p < 0.05, **p < 0.01, ****p < 0.0001.

To obtain the cell populations of interest, we first isolated CD4^+^ T cells from human peripheral blood. These cells were flow cytometrically sorted based on a CD25^lo^CD127^hi^CD45RA^+^GPA33^int^ phenotype to obtain naïve Tconv cells, or a CD25^hi^CD127^lo^CD45RA^+^GPA33^hi^ phenotype to obtain naïve Treg cells, as described before^21,31^ (Supplementary Fig. 1). After sorting, the cells were expanded using agonistic monoclonal antibodies (mAbs) against CD3 and CD28 in the presence of IL-2. Cells derived from naïve Treg cells are hereinafter referred to as tTreg cells. To generate iTreg cells, TGF-β was added to Tconv cell cultures. After 2 weeks of expansion in vitro, cells “rested” for 4 days prior to restimulation with anti-CD3/CD28 antibodies or not for 24 h and analysis (Fig. 1a).

At this point, Tconv cells were FOXP3^–^Helios^–^ and largely retained this phenotype after CD3/CD28-mediated restimulation (Fig. 1b,c). tTreg cells showed a uniform expression of FOXP3 and Helios (IKFZ2), both before and after restimulation, suggesting that the isolation and cell culture method generated pure tTreg cells (Fig. 1b,c). The iTreg cells showed bimodal FOXP3 expression, with an upregulation of FOXP3 upon CD3/CD28-mediated restimulation, while Helios was minimally expressed, as described^7^ (Fig. 1b,c).

The TSDR of the *FOXP3* gene locus had the highly demethylated configuration in tTreg cells and was methylated in Tconv and iTreg cells, in agreement with published data^5,32^ (Fig. 1d). Next, we functionally characterized the cell populations in a standard assay, testing their ability to suppress CD3-induced proliferation of CellTrace Violet-labeled Tconv cells from peripheral blood. In this assay, tTreg cells were suppressive and more so than iTreg cells, while Tconv cells were not, in agreement with earlier research^33^ (Fig. 1e). Together, these data validate the purification and cell culture protocols to generate tTreg, iTreg and Tconv cell populations for further characterization.

### Expression of inhibitory and stimulatory receptors on tTreg and iTreg cells

**Figure 2:**
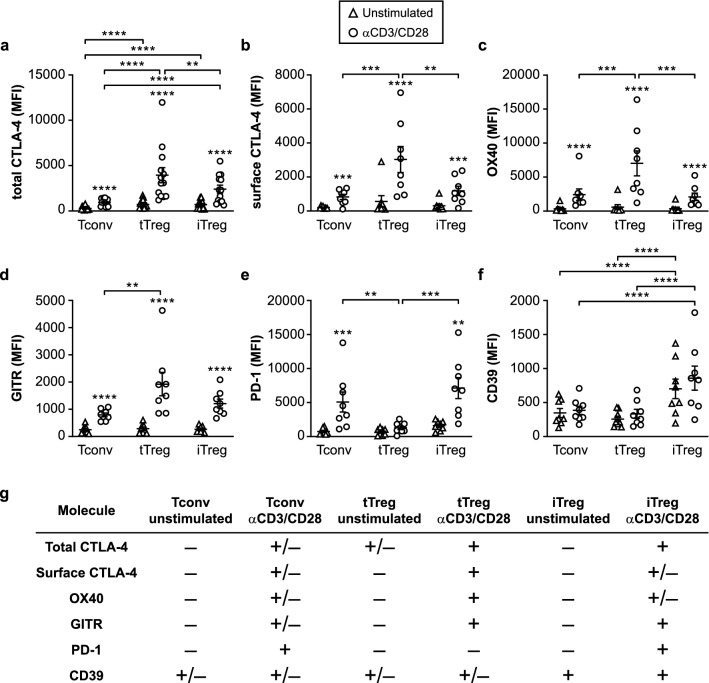
Expression of characteristic cell surface molecules associated with Treg cells. **(a–f)** Quantification of flow cytometric analysis of the protein expression of total CTLA-4 (cell surface plus intracellular) (*n* = 13) **(a)**, surface CTLA-4 (*n* = 8) **(b)**, surface OX40 (*n* = 8) **(c)**, surface GITR (*n* = 8) **(d)**, surface PD-1 (*n* = 8) **(e)** and surface CD39 (ENTPD1) (*n* = 8) **(f)** using indicated cell populations. Mean fluorescence intensity (MFI) is depicted on the y-axis. Two-way ANOVA with Tukey’s post hoc test was used for statistical analysis. Data are presented as mean ± SEM. Sample size (*n*) represents cells from individual donors, analyzed in independent experiments. Asterisks without lines indicate comparisons within cell types following restimulation. *p < 0.05, **p < 0.01, ***p < 0.001, ****p < 0.0001. **(g)** Table summarizing the data in **(a–f)**, indicating relatively low (−), intermediate (+/−) or high (+) expression levels.

We next examined the expression of a number of important inhibitory and stimulatory cell surface receptors. Among these, CTLA-4 is a key mediator of suppression by tTreg cells^34^ by reducing the availability of CD80/86 for the costimulatory receptor CD28 on Tconv cells^35,36^. As CTLA-4 recycles between the cell surface and endosomes^37^, we measured both total (cell surface plus intracellular) CTLA-4 levels, as well as specifically the CTLA-4 levels on the cell surface only. Both tTreg and iTreg cells showed higher total CTLA-4 expression than Tconv cells (Fig. 2a, Supplementary Fig. 2). All three cell types upregulated total CTLA-4 protein levels upon CD3/CD28-mediated restimulation, but levels were still higher in tTreg and iTreg cells than in Tconv cells. Cell surface expression of CTLA-4 was only found on all three cell types after CD3/CD28-mediated restimulation, with tTreg cells showing the highest levels (Fig. 2b, Supplementary Fig. 2). The costimulatory receptors OX40 and GITR have received attention as regulators of Treg cell responses^38–40^. They were only detected on the cell surface following CD3/CD28-mediated restimulation, on all three cell types, with highest levels on tTreg cells (Fig. 2c,d, Supplementary Fig. 2). PD-1, which is described as a coinhibitory receptor for both Tconv and Treg cells^41^, was induced by CD3/CD28-mediated restimulation on both iTreg and Tconv cells, but not on tTreg cells (Fig. 2e, Supplementary Fig. 2). The cell surface enzyme CD39 (ENTPD1) facilitates extracellular ATP hydrolysis, thereby generating adenosine that has immunosuppressive effects on Tconv cells^42^. CD39 protein levels were highest on iTreg cells both before and after restimulation (Fig. 2f, Supplementary Fig. 2). This analysis shows that tTreg cells differ from Tconv and iTreg cells particularly in high CTLA-4 cell surface expression and lack of PD-1 expression after CD3/CD28-mediated activation, while iTreg cells are unique in high CD39 expression (Fig. 2g).

### Proteomic analysis reveals that iTreg cells are distinct from tTreg and Tconv cells

**Figure 3:**
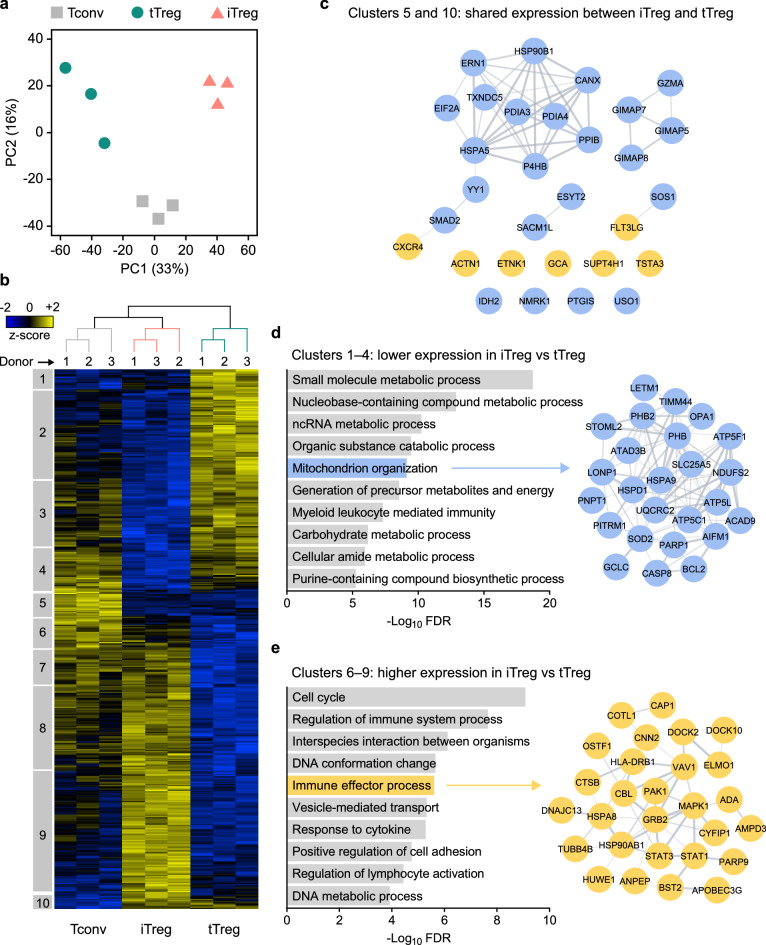
The iTreg, tTreg and Tconv cell proteomes are distinct. **(a)** PCA plot of the proteome of unstimulated Tconv, tTreg and iTreg cells (*n* = 3). **(b)** Heat map showing hierarchical clustering of the 475 proteins that were differentially expressed between unstimulated Tconv, tTreg and iTreg cells (ANOVA, FDR < 0.05). Samples are colored according to cell type as in **(a)**. Z-scores showing relative protein expression values are color-coded. Numbered boxes (1–10) indicate clusters of proteins. **(c)** STRING network of proteins that have shared low (cluster 5) or high (cluster 10) expression in iTreg and tTreg cells, relative to Tconv cells. Proteins with high or low expression are depicted as yellow or blue nodes, respectively. Protein associations according to STRING are connected with lines. **(d)** GO enrichment analysis of clusters 1–4 containing proteins with low expression in iTreg cells compared to tTreg cells, showing ten significantly enriched GO biological processes. For example, proteins associated with mitochondrion organization are shown in a STRING network. Blue nodes indicate low expression in iTreg cells relative to tTreg cells. **(e)** GO enrichment analysis of clusters 6–9 containing proteins with high expression in iTreg cells compared to tTreg cells, showing ten significantly enriched GO biological processes. For example, proteins involved in immune effector processes are displayed in a STRING network. Yellow nodes indicate high expression in iTreg cells relative to tTreg cells.

We performed label-free quantitative proteomics to compare overall protein expression profiles in resting Tconv, tTreg and iTreg cells in an unbiased manner. Based on the levels of the 4285 proteins that were identified (Supplementary Table 1), principal component analysis (PCA) showed distinct grouping of Tconv, tTreg and iTreg cells (Fig. 3a). PC1 explained most of the differences between the populations (33%). It revealed that iTreg and tTreg cells are distinct from one another and each more similar to Tconv cells. In contrast, PC2 (16%) indicated commonalities between iTreg and tTreg cells that set them apart from Tconv cells. Hierarchical clustering, based on the 475 proteins that were differentially expressed between the three cell types, also revealed marked differences between all populations (Fig. 3b, Supplementary Table 2). Each cell population exhibited a unique pattern of protein clusters with relatively high or low expression. This analysis clustered iTreg and Tconv cells together in one branch of the dendrogram, whereas tTreg cells clustered in a different branch, showing that iTreg cells are overall more related to Tconv cells than to tTreg cells. In fact, only two small protein clusters were shared by iTreg and tTreg cells but not Tconv cells: cluster 5 and cluster 10, which represented low or high expression of proteins compared to Tconv cells, respectively. Gene Ontology (GO) analysis indicated an overrepresentation of proteins associated with protein folding and endoplasmic reticulum stress in these two clusters (Fig. 3c, Supplementary Fig. 3). Notable was furthermore shared low expression of GIMAP proteins that are implicated in T cell homeostasis^43^ and shared low expression of YY1, which inhibits FOXP3 expression and function^44^.

Apart from this limited overlap in protein expression between iTreg and tTreg cells, a far greater number of proteins (clusters 1–4 and 6–9) was differentially expressed between these cell types (Fig. 3b). Proteins in clusters 1–4 were expressed at lower levels in iTreg cells than in tTreg cells and were mainly associated with metabolic processes (Fig. 3d). Part of these clusters showed relatively low protein expression in both iTreg and Tconv cells (clusters 1–2), whereas the other part showed low protein expression uniquely in iTreg cells (clusters 3–4) (Supplementary Fig. 3). Noteworthy was the lower expression of 26 proteins involved in mitochondrion organization in iTreg cells compared to tTreg cells (Fig. 3d). Of these, 18 proteins were located in cluster 2, indicating that iTreg cells largely shared low expression of these proteins with Tconv cells. Clusters 6–9 consisted of proteins that were present at higher levels in iTreg cells than in tTreg cells. These proteins are mainly involved in cell cycling, immune effector processes, vesicle-mediated transport and cell adhesion (Fig. 3e). These clusters included proteins related to lymphocyte activation, such as MHC class II, STAT transcription factors, and signaling molecules such as CBL, VAV1, GRB2, MAPK1 and PAK1 (Fig. 3e). High expression of these proteins was partly shared by iTreg and Tconv cells (clusters 6–8) and partly unique for iTreg cells (cluster 9) (Supplementary Fig. 3). This analysis indicates that iTreg cells display a protein expression profile that largely sets them apart from tTreg cells and indicates similarity with Tconv cells particularly in metabolic features and potential responses to activation signals. However, iTreg cells do share some features with tTreg cells, in particular regarding endoplasmic reticulum-associated processes.

We also analyzed the proteomes of Tconv, tTreg and iTreg cells following CD3/CD28-mediated restimulation (Supplementary Table 1). Distinct clusters were identified by PCA, showing that particularly Tconv and iTreg cells altered their protein expression upon activation (Supplementary Fig. 4a). A much smaller response was observed in tTreg cells, as these samples clustered adjacent to each other regardless of stimulation. We performed gene set enrichment analysis (GSEA) to study the GO biological processes associated with the changes in protein expression following restimulation. GSEA emphasized the different responses between the cell types, indicating that iTreg cells, like Tconv cells and unlike tTreg cells, strongly responded to CD3/CD28-mediated restimulation, for example regarding processes associated with cell division (Supplementary Fig. 4b).

### A core signature reflecting unique Treg cell properties is present in tTreg but not iTreg cells

**Figure 4:**
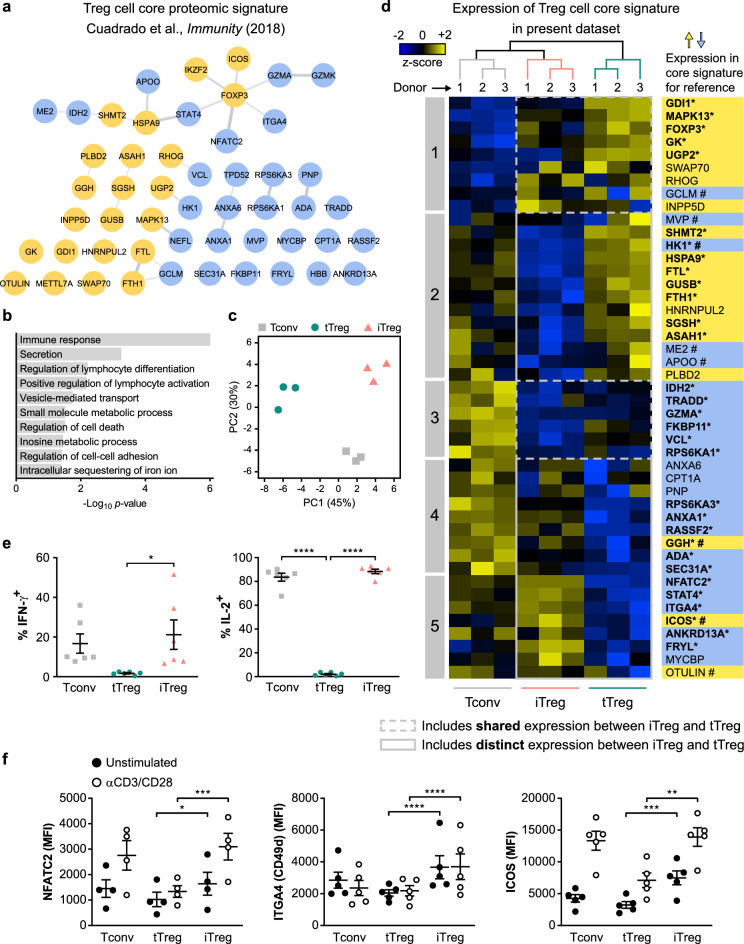
A Treg cell core signature is present in in vitro-expanded tTreg but not iTreg cells. **(a)** STRING network displaying 51 proteins of a previously defined Treg cell core proteomic signature, which represents high (yellow) or low (blue) protein expression in ex vivo naïve and effector Treg cells compared to other CD4^+^ T cells from human blood^22^. **(b)** GO enrichment analysis of the Treg cell core signature proteins, showing ten significantly enriched GO biological processes. **(c)** PCA plot based on the expression of Treg cell core signature proteins in unstimulated Tconv, tTreg and iTreg cells in the present dataset (*n* = 3). **(d)** Heat map showing hierarchical clustering of 45 proteins of the Treg cell core signature based on their expression in unstimulated Tconv, tTreg and iTreg cells. Samples are colored according to cell type as in **(c)**. Z-scores depicting relative protein expression values are color-coded. Numbered boxes (1–5) indicate clusters of proteins. For reference, protein names are colored according to high (yellow) or low (blue) expression in the Treg cell core signature as shown in **(a)**. Significantly differential expression (FDR < 0.05) is indicated by asterisks and bold letters. Proteins are marked (#) when expression in in vitro-expanded tTreg cells from the present dataset deviates from the core signature derived from ex vivo Treg cells^22^. **(e)** Quantification of flow cytometric analysis of intracellular IFN-γ and IL-2 protein expression in indicated PMA/ionomycin-treated cell populations from the same donors (*n* = 6). Percentage of IFN-γ^+^ or IL-2^+^ cells is shown on the y-axis. One-way ANOVA with Tukey’s post hoc test was used for statistical analysis. **(f)** Quantification of flow cytometric analysis of the protein expression of total NFATC2 (*n* = 4), surface ITGA4 (CD49d) (*n* = 5) and surface ICOS (*n* = 5) on indicated cell populations. MFI is depicted on the y-axis. Two-way ANOVA with Tukey’s post hoc test was used for statistical analysis. Data are presented as mean ± SEM. Sample size (*n*) represents cells from individual donors, analyzed in independent experiments. *p < 0.05, **p < 0.01, ***p < 0.001, ****p < 0.0001.

Recently, we performed proteomic analysis of Treg cells that were isolated to high purity in the naïve state from human peripheral blood and therefore very likely represent tTreg cells. In that study, we defined a proteomic signature shared between these naïve tTreg cells and effector phenotype Treg cells that distinguishes them from CD4^+^ Tconv cells, which we termed the Treg cell core signature^22^. This signature defines characteristic high or low expression in Treg cells compared to Tconv cells of certain proteins that remains unaltered upon cell activation. These proteins indicate stable differences between Treg and Tconv cells in specific aspects of signal transduction, transcriptional regulation, iron storage, lysosomal processes and cell metabolism (Fig. 4a,b). Here, we questioned whether the Treg cell core signature is also present in iTreg cells.

In our proteomics dataset, we detected 45 out of 51 proteins of the Treg cell core signature and evaluated their expression in expanded Tconv, tTreg and iTreg cells (Supplementary Table 3). PCA indicated distinct clustering of these three cell types based on Treg cell core signature protein levels, with iTreg cells clearly clustering away from tTreg cells (Fig. 4c). Hierarchical clustering based on this signature also separated the three cell types (Fig. 4d). The Treg cell core signature, with some exceptions, was preserved in tTreg cells following in vitro expansion, as reported previously^22^ (Fig. 4d). tTreg and iTreg cells shared a number of the Treg cell core signature proteins with characteristic high (cluster 1—including FOXP3, as expected) or low (cluster 3) expression levels relative to Tconv cells. Moreover, iTreg cells had characteristic low expression of the core signature proteins MVP, HK1, ME2 and APOO and high expression of ICOS, while in vitro-expanded tTreg cells did not. However, most Treg cell core signature proteins were not expressed at the characteristic levels in iTreg cells.

For instance, iTreg cells expressed high levels of the transcription factor STAT4, while low levels in Treg cells are critical to conserve Treg cell function^22^. Low expression of transcription factor NFATC2 is another feature of Treg cells^22^, but iTreg cells shared relatively high expression of this protein with Tconv cells. STAT4 and NFATC2 are mediators of pro-inflammatory cytokine signaling and regulate *IFNG* and *IL2* gene expression, respectively^45,46^. We observed that iTreg cells but not tTreg cells were able to produce IFN-γ and IL-2 following PMA/ionomycin-mediated stimulation, suggesting that iTreg cells lack anti-inflammatory characteristics of Treg cells (Fig. 4e, Supplementary Fig. 5a). We furthermore confirmed differential expression of core signature proteins NFATC2, ITGA4 and ICOS between tTreg and iTreg cells by flow cytometry, both before and after CD3/CD28-mediated restimulation (Fig. 4f, Supplementary Fig. 5b).

Since iTreg cells responded strongly to CD3/CD28-mediated restimulation in terms of altered protein expression (Supplementary Fig. 4a,b), we examined whether they obtained more of the Treg cell core signature under these conditions. The core signature is present in naïve and effector tTreg cells ex vivo, and therefore, the expression of core signature proteins remains rather stable in tTreg cells upon activation^22^. This was indeed observed in restimulated tTreg cells, and interestingly, levels of their own core signature proteins were also largely maintained in iTreg or Tconv cells upon restimulation (Supplementary Fig. 4c, Supplementary Table 3). Thus, iTreg cells did not have characteristic expression of many Treg cell core signature proteins, regardless of whether the cells were resting or had been restimulated via CD3/CD28. These data suggest that iTreg cells share only a part of the unique characteristics of ex vivo Treg cells.

### iTreg cells have aspects of an effector Treg cell signature

**Figure 5:**
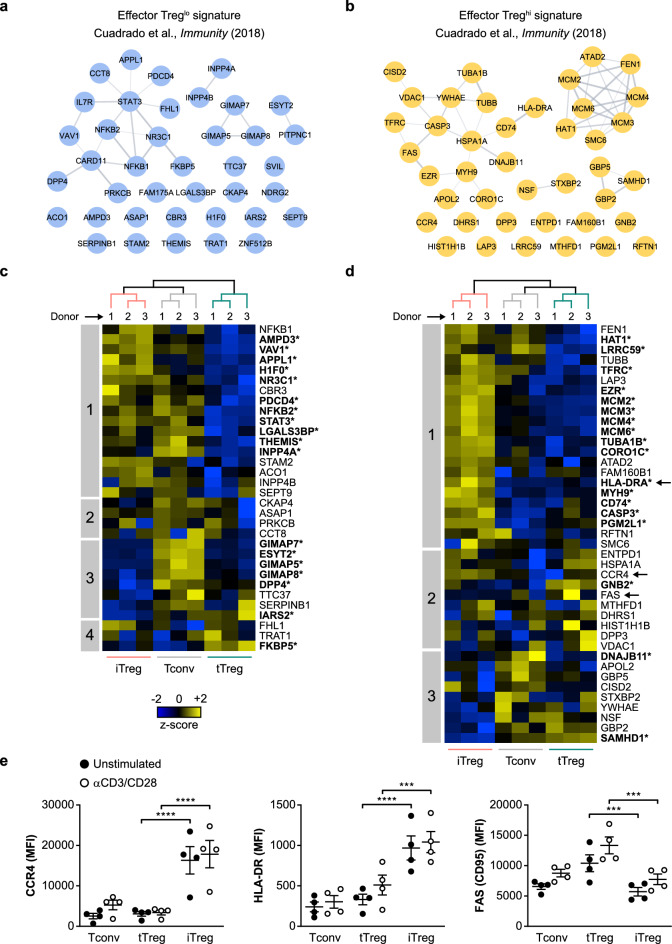
Aspects of an effector Treg cell signature are present in iTreg cells. **(a,b)** The effector Treg cell signature represents 39 lowly **(a)** and 41 highly expressed proteins **(b)** in ex vivo effector Treg cells compared to naïve Treg cells and other CD4^+^ T cells from human blood^22^. Shown are STRING networks displaying the lowly and highly expressed proteins as blue and yellow nodes, respectively. **(c,d)** Heat maps showing hierarchical clustering of the lowly **(c)** or highly **(d)** expressed proteins of the effector Treg cell signature based on their expression in unstimulated Tconv, tTreg and iTreg cells (*n* = 3). Samples are colored according to cell type. Z-scores showing relative protein expression values are color-coded. Numbered boxes indicate clusters of proteins. Significantly differential expression is indicated by asterisks and bold letters (FDR < 0.05). CCR4, HLA-DRA and FAS (CD95) are marked in **(d)**, as these molecules are also analyzed by flow cytometry in **(e)**. **(e)** Quantification of flow cytometric analysis of the protein expression of surface CCR4 (*n* = 4), surface HLA-DR (*n* = 4) and surface FAS (*n* = 4) on indicated cell populations. MFI is depicted on the y-axis. Two-way ANOVA with Tukey’s post hoc test was used for statistical analysis. Data are presented as mean ± SEM. Sample size (*n*) represents cells from individual donors, analyzed in independent experiments. ***p < 0.001, ****p < 0.0001.

We previously also defined an effector Treg cell signature, composed of proteins that set ex vivo effector Treg cells apart from naïve Treg cells and Tconv cells^22^. The 39 lowly expressed proteins of this signature (effector Treg^lo^) primarily include those associated with signaling by the TCR/CD3 complex and CD28 (e.g. THEMIS, TRAT1, VAV1), phosphatidylinositol (e.g. INPP4A, INPP4B), NF-κB (e.g. NFKB1, NFKB2) and JAK-STAT (e.g. STAT3, IL7R) (Fig. 5a). The 41 highly expressed proteins of the effector Treg cell signature (effector Treg^hi^) are predominantly associated with DNA replication (e.g. MCM proteins), apoptosis (e.g. FAS, CASP3), vesicular trafficking (e.g. STXBP2, NSF) and cytoskeletal reorganization (e.g. TUBA1B, TUBB) (Fig. 5b). Here, we examined the effector Treg cell signature in in vitro-expanded Tconv, tTreg and iTreg cells and questioned whether iTreg cells acquire effector Treg cell-like properties.

Among the 32 proteins of the effector Treg^lo^ signature detected, a limited number (clusters 3–4) exhibited characteristic low expression in iTreg cells (Fig. 5c, Supplementary Table 4). Proteins in cluster 3 were also expressed at low levels in tTreg cells and included DPP4 (CD26) and members of the GIMAP family. However, 17 out of 32 effector Treg^lo^ signature proteins did not have the characteristic low expression in iTreg cells, whereas they did in tTreg cells (cluster 1). These included important transcription factors, such as NFKB1, NFKB2, and STAT3 and signaling molecules such as VAV1, STAM2, INPP4A and THEMIS.

All 41 proteins of the effector Treg^hi^ signature were detected and in iTreg cells 22 out of 41 proteins exhibited characteristic high expression (cluster 1) as opposed to in tTreg and Tconv cells (Fig. 5d, Supplementary Table 4). These proteins included MHC class II and were furthermore primarily involved in DNA replication (e.g. MCM proteins) and cytoskeletal processes such as cytokinesis (e.g. MYH9, CORO1C), indicating cell proliferation. Additionally, iTreg cells had high expression of some other signature proteins, including the cell surface receptors ENTPD1 (CD39) and CCR4 (cluster 2), but this varied more between samples. For CD39 we already demonstrated uniquely high cell surface expression on iTreg cells by flow cytometry (Fig. 2f). We also demonstrated by flow cytometry that CCR4 and HLA-DR were highly expressed on iTreg cells, whereas FAS (CD95) was highly expressed on tTreg cells, in agreement with the results from the proteome measurements (Fig. 5e, Supplementary Fig. 6). Altogether, the majority of effector Treg^hi^ signature proteins was expressed at relatively high levels in iTreg cells. Thus, the effector Treg cell signature is partially present in iTreg cells, indicating that iTreg cells resemble ex vivo effector Treg cells to some extent.

## Discussion

The Treg cell lineage can be subdivided into tTreg and pTreg cells based on developmental origin^15,47^. tTreg cells have escaped negative selection in the thymus and are mainly geared towards self-antigen recognition^15^. They comprise the majority of Treg cells in vivo and control systemic and tissue-specific autoimmunity^48,49^. pTreg cells recognize foreign antigen and are described to reside at mucosal surfaces and the maternal–fetal interface^14,50^. Using the cell surface marker Nrp1, pTreg cells can be distinguished from tTreg cells in mice, where common and unique features in their phenotypes and transcriptome profiles have been observed^11,12^. tTreg and pTreg cells complement each other in immune homeostasis^51,52^, which may in part be due to their distinct TCR repertoires and differential expression of transcriptional regulators^14,53^. In human, effector phenotype tTreg or pTreg cells cannot be discriminated for lack of surface markers. Because pTreg cells cannot be isolated ex vivo, TGF-β-driven iTreg cells are often employed as an in vitro model for Treg cell function, differentiation or metabolism.

Upon their generation from Tconv cells, iTreg cells acquire Treg cell-like features such as expression of FOXP3 and CTLA-4^28^. However, FOXP3 expression in iTreg cells is heterogeneous, as observed by us and others^24,25,54,55^. Stable FOXP3 expression is established at the epigenetic level by demethylation of several regions within the *FOXP3* gene locus. A critical region for sustained *FOXP3* expression is the TSDR in the CNS2 enhancer, which we confirmed to be highly demethylated in tTreg cells but not in iTreg cells, as described^5,15^. TGF-β can induce *FOXP3* expression in activated Tconv cells via the CNS1 enhancer, which is reportedly less stable^15^. Natural compounds including all-trans retinoic acid (ATRA)^56^, a vitamin A metabolite, and microbial product butyrate^57,58^ can enhance FOXP3 expression. In vivo, these compounds are involved in pTreg cell generation, particularly in the gut. Schmidt et al. reported that addition of ATRA or butyrate to TGF-β increased the frequency of human FOXP3-expressing cells, but nevertheless did not improve CTLA-4 expression and suppressive function^55^. It was recently reported for murine iTreg cells that deprivation of CD28 costimulation led to increased DNA demethylation within Treg cell-specific genes, even including *Foxp3* CNS2 demethylation, giving rise to more stable Foxp3 expression and enhanced suppressive function^59^. It will therefore be of interest to generate human iTreg cells in absence of CD28 costimulation and to determine whether such cells resemble tTreg cells better than the iTreg cells generated by the more traditional protocol used here.

In a comprehensive proteomic analysis, Schmidt et al. found TGF-β to be the key driver of the iTreg cell phenotype that was not majorly affected by ATRA or butyrate^60^. Addition of rapamycin to TGF-β and ATRA generated iTreg cells with a unique protein expression profile and enhanced suppressive activity in vitro, but these iTreg cells were not suppressive in vivo^55,60^. Given those data, we here used iTreg cells induced by TGF-β only and compared them to highly pure GPA33^hi^ tTreg cells^21,22^ and Tconv cells, using several benchmarks of ex vivo Treg cells as previously defined by proteomics^22^. Proteomics gives more direct insight into the functional cellular programs compared to transcriptomics due to differential regulation of protein and mRNA levels, as also shown for Treg cells^22,60,61^.

iTreg cells proved to display a protein expression profile that markedly sets them apart from tTreg cells. The proteins that were differentially expressed between iTreg and tTreg cells were enriched in metabolic regulators (Fig. 3d). Studies on T-cell metabolism have used iTreg cells as representative of ex vivo Treg cells and described certain metabolic features that would be unique to Treg cells as compared to CD4^+^ Tconv cells^24,25,54^. The clear distinction in expression of metabolic regulators between iTreg and tTreg cells underlines that these cell populations should not be equated in metabolic studies. iTreg cells furthermore differed from tTreg cells in high expression of signaling proteins involved in lymphocyte activation. This result predicts a divergent response of iTreg versus tTreg cells to extracellular stimuli. Based on the proteomic Treg cell core signature, we have previously reported that tTreg cells have adaptations in signaling pathways downstream of immune receptors such as the TCR, CD28 and cytokine receptors^22^. In particular, low expression of signaling molecules in tTreg cells may lead to attenuated responses to pro-inflammatory cytokines, as we demonstrated before for STAT4^22^. The high expression of multiple signaling molecules in iTreg cells, together with proteins involved in the cell cycle indicates a more activated and responsive state of iTreg cells compared to tTreg cells. Accordingly, we observed that CD3/CD28-mediated restimulation induced a strong response in iTreg and Tconv cells. In contrast, tTreg cells had a strikingly modest response to this stimulus. The proteomic similarities between iTreg and tTreg cells were limited. GO analysis primarily indicated a similar enrichment of proteins involved in endoplasmic reticulum-associated processes. The transcription factor Eos (Ikzf4) is associated with controlling Treg cell function and phenotype and interacts directly with Foxp3 to mediate gene silencing in murine Treg cells^62^. EOS is also reported to be highly expressed in human iTreg cells^60^, which we confirmed by proteomics and flow cytometry (Supplementary Fig. 7). We furthermore observed shared low expression of transcription factor YY1 by proteomics, which can also interact with FOXP3 to regulate transcription^44,63^. Helios was not expressed at similar high levels in iTreg cells as observed in tTreg cells, but minor upregulation was found compared to Tconv cells. It has been demonstrated in mice that, after recent egress from the thymus, a small proportion of naïve CD4^+^ Tconv cells can develop into Helios^+^ pTreg cells^64^. Nevertheless, Helios is preferentially expressed in tTreg cells and marks stable commitment to the human Treg cell lineage^7,8^.

Upon activation, tTreg cells can travel from secondary lymphoid organs through the blood to peripheral tissues, where they experience environmental signals. They can adapt themselves to locally resident effector T cells by responding to specific cytokines^49^. This involves the gain of lineage-determining transcription factors such as GATA-3 or T-bet in addition to FOXP3, which alters their functionality. Regardless of their activation status, tTreg cells retain core features that safeguard their suppressive nature and prevent them from exerting pro-inflammatory functions^22^. To establish whether in vitro-generated iTreg cells acquire such features, we examined to what extent they exhibited the characteristic proteomic signatures from human Treg cells that were analyzed directly from peripheral blood^22^. The first benchmark we used was the Treg cell core signature, a conserved set of proteins between naïve and effector Treg cells compared to other CD4^+^ T cell subsets that emphasizes critical Treg cell aspects^22^. This signature strongly suggests differential wiring of signaling pathways in Treg and Tconv cells, given the constitutive differential expression of key signal transduction components between these cell types. We observed that iTreg cells only share a limited part of these conserved characteristics, while the signature was largely present in the in vitro-expanded tTreg cells studied. Noteworthy, expression of the core signature proteins remained comparable upon CD3/CD28-mediated restimulation in all cell types studied, supporting that the signature reflects essential Treg cell properties. Thus, iTreg cells do not resemble tTreg cells found in peripheral blood. Since pTreg cells cannot be isolated ex vivo due to lack of cell surface markers, it cannot be directly assessed to what extent iTreg cells share features with pTreg cells, whose TGF-β-induced features they are thought to reproduce^14^.

Based on the differential expression of signaling molecules, it is likely that iTreg cells show an enhanced response to pro-inflammatory cytokines compared to tTreg cells. For example, the Treg cell core signature includes low expression of STAT4 and NFATC2, which are mediators of pro-inflammatory cytokine signaling^45,46^. Low expression of these molecules may aid tTreg cells to protect their anti-inflammatory nature in a pro-inflammatory environment, while they can still respond to pro-inflammatory cytokines through other pathways^22^. We have previously shown that deliberate elevation of STAT4 expression in tTreg cells increases their susceptibility to destabilization by pro-inflammatory cytokines, provoking effector cytokine production and loss of FOXP3 expression^22^. Both STAT4 and NFATC2 had relatively high expression in iTreg cells compared to tTreg cells, suggesting that iTreg cells may respond to pro-inflammatory cues in a pro-inflammatory manner, e.g. by IFN-γ production^65^. iTreg cells were shown to be less stable after transfer into inflamed mice than tTreg cells^26^. This has been attributed to their fragile epigenetic stability, but our findings now suggest that this may also be caused by their high expression of involved mediators, such as STAT4 and NFATC2.

The second benchmark we examined was an effector Treg cell signature, which sets effector Treg cells apart from naïve Treg cells and other CD4^+^ T cell subsets^22^. Relative to protein expression in tTreg and Tconv cells, iTreg cells acquired aspects of the effector Treg cell signature, indicating that iTreg cells may resemble ex vivo effector Treg cells to some extent. Since iTreg cells are thought to mimic pTreg cells because of TGF-β-mediated induction, the enrichment of the effector Treg cell signature in iTreg cells may fit with the idea that the effector Treg cell population in peripheral blood contains both activated tTreg cells and pTreg cells^66,67^.

In conclusion, our global proteomic analysis showed that iTreg and tTreg cells are distinct and each share more protein expression with Tconv cells than with one another, providing a resource for further research into human Treg cells. Moreover, iTreg cells only partially have the protein expression features of ex vivo effector Treg cells. These aspects limit their utility as an in vitro model for Treg cells.

## Methods

### Cell isolation and flow cytometric sorting

Human materials were obtained in compliance with the Declaration of Helsinki and the Dutch rules regarding the use of human materials from voluntary donors. Buffy coats from healthy anonymized male donors were obtained after their written informed consent, as approved by the Ethical Advisory Council of Sanquin Blood Supply Foundation, Plesmanlaan 125, Amsterdam, the Netherlands. Total CD4^+^ T cells were collected from buffy coats by first performing a Ficoll-Paque Plus (GE Healthcare) density gradient centrifugation to isolate PBMCs and subsequently using CD4 magnetic MicroBeads (Miltenyi Biotec) according to the manufacturers’ protocols. Alternatively, total CD4^+^ T cells were directly isolated from buffy coats using the StraightFrom Buffy Coat CD4 MicroBead kit (Miltenyi Biotec) according to the manufacturer’s instructions. To sort naïve Tconv and tTreg cells, CD4^+^ T cells were stained with CD4-PE-Cy7, CD127-BV421 (BioLegend), CD25-PE (BD Biosciences), CD45RA-FITC (Immunotools) and GPA33-AF647^68^ mAbs as indicated (Supplementary Fig. 1). Cells were sorted on a MoFlo Astrios using Summit software version 6.2 (Beckman Coulter) or BD FACS Aria II using FACSDiva software version 8 (BD Biosciences). Either propidium iodide (Sigma) or near-IR dead cell stain kit (Invitrogen) was used as a live/dead marker.

### T-cell expansion cultures and restimulation

Following cell sorting, human naïve Tconv and tTreg cells were cultured in 96-well round bottom plates (Greiner) at 1 × 10^4^ cells per well using IMDM (Gibco, Life Technologies) supplemented with 8% FCS (Sigma), penicillin/streptomycin (Roche) and 300 IU/ml IL-2 (DuPont Medical), hereinafter referred to as T-cell medium, at 37 °C and 5% CO_2_. Cells were expanded using agonistic mAbs against CD3 (clone CLB-T3/4.E, IgE isotype, Sanquin, 0.1 μg/ml) and CD28 (clone CLB-CD28/1, Sanquin, 0.2 μg/ml) in solution, added on day 0 and 7 during expansion cultures. On day 4, fresh T-cell medium was added. From day 7 to day 14, cells were cultured in 24-well plates (Greiner) at 5 × 10^5^ cells per ml, while fresh T-cell medium was added on day 11. On day 14, cells were cultured in 6-well plates (Corning) at 1 × 10^6^ cells per ml for 4 days using fresh T-cell medium in the absence of agonistic mAbs. To generate iTreg cells, naïve Tconv cells were cultured as described above in the presence of TGF-β (Peprotech, 10 ng/ml). Prior to restimulation experiments, dead cells were removed by Ficoll-Paque Plus density gradient centrifugation. T cell restimulation was performed where indicated, using T-cell medium in the presence of agonistic mAbs against CD3 (0.1 μg/ml) and CD28 (0.2 μg/ml), but without TGF-β. For cytokine production assays, resting T cells were cultured for 5 h in presence of GolgiPlug (BD Biosciences, 1:1000 dilution), with or without PMA (5 ng/ml) and ionomycin (750 ng/ml).

### Flow cytometry

For expression analysis of cell surface molecules, T cells were washed in PBS/1% FCS and stained using the following mAbs in appropriate combinations: anti-OX40-PE-Cy7 (BioLegend), anti-GITR-BV421 (BioLegend), anti-PD-1-APC-Cy7 (BioLegend), anti-CD39-BV510 (BioLegend), anti-ITGA4-PE (CD49d) (BD Biosciences), anti-ICOS-PerCP-Cy5.5 (BioLegend), anti-CCR4-PE-Cy7 (BioLegend), anti-HLA-DR-APC-eFluor 780 (Invitrogen), anti-HLA-DR-BV605 (BD Biosciences) and anti-FAS-BB700 (CD95) (BD Biosciences). Cell surface expression of CTLA-4 was measured by adding anti-CTLA-4-PE-Dazzle594 mAb (BioLegend) in culture during restimulation for 24 h. Near-IR dead cell stain kit (Invitrogen) was used as a live/dead marker. For intracellular staining, cells were fixed and permeabilized using the FOXP3 transcription factor staining buffer set (Invitrogen) according to the manufacturer’s instructions. Next, cells were stained with combinations of anti-FOXP3-APC (Invitrogen), anti-Helios-PE-Cy7, anti-CTLA-4-PE-Dazzle594, anti-EOS-PE (BioLegend) and anti-NFATC2-AF488 (Cell Signaling Technology) mAbs. Anti-IL-2-PerCP-Cy5.5 (BioLegend) and anti-IFN-γ-BV421 (BD Biosciences) mAbs were used for intracellular cytokine staining. Flow cytometry was performed using a BD LSR Fortessa or BD LSR II cell analyzer (BD Biosciences). Sample acquisition was performed using FACSDiva software version 8 and data were analyzed using FlowJo software version 10.6.0.

### Methylation assay of the TSDR

The methylation status of the TSDR within the *FOXP3* locus was determined in Tconv, tTreg and iTreg cells as described previously^31^. Only male donors were used for these analyses, given the X-chromosomal location of the *FOXP3* locus. In brief, expanded T cells were resuspended in PBS for proteinase K digestion, followed by bisulfite conversion of DNA using the EZ DNA Methylation-Direct kit (Zymo Research) and methylation-specific quantitative PCR using iQTM SYBR Green Supermix (Bio-Rad)^69^. Methylation-specific primers were 5ʹ-CGATAGGGTAGTTAGTTTTCGGAAC-3ʹ and 5ʹ-CATTAACGTCATAACGACCGAA-3ʹ. Demethylation-specific primers were 5ʹ-TAGGGTAGTTAGTTTTTGGAATGA-3' and 5ʹ-CCATTAACATCATAACAACCAAA-3ʹ. Melt curve analysis was performed on a LightCycler^®^ 480-II (Roche). Methylation of the TSDR (%) was calculated using the following formula: 100/(1 + 2^Ct[CG] − Ct[TG]^), where Ct[CG] is defined as Ct values obtained using methylation-specific primers and Ct[TG] is defined as Ct values obtained using demethylation-specific primers.

### Suppression assay

Total human PBMCs were washed and resuspended in PBS, followed by incubation for 8 min using 5 μM CellTrace Violet dye (Invitrogen). Following fluorescent labeling, an equal volume of FCS was added and cells were subsequently washed twice in IMDM supplemented with 8% FCS. CellTrace Violet-labeled PBMCs were cocultured with expanded Tconv, tTreg or iTreg cells for 96 h at indicated ratios, in the presence of anti-CD3 mAb (0.05 μg/ml). Proliferation of PBMCs was examined by measuring CellTrace Violet dye dilution by flow cytometry on a BD LSR Fortessa or BD LSR II cell analyzer. Data were analyzed using FlowJo software (version 10.6.0).

### Sample preparation for mass spectrometry

Following T-cell expansion, at least 1 × 10^6^ resting or restimulated Tconv, tTreg and iTreg cells were washed in PBS and lysed in 100 mM Tris HCl pH 8.0 with 4% SDS and 100 mM DTT, as described previously^22^. Samples were heated at 95 °C, sonicated (Bioruptor) and centrifuged (16,000×*g*), followed by isolation of cell lysates and storage at − 80 °C. Cell lysates were processed using filter-aided sample preparation (FASP) as described^70^. In short, proteins were alkylated and digested into peptides using sequencing-grade Trypsin (Promega). Samples were desalted using StageTips^71^, the aqueous phase was evaporated in a speedvac, and proteins were reconstituted in 2% acetonitrile in 0.1% TFA in water before analysis by mass spectrometry.

### Mass spectrometry data acquisition

As described previously^22^, tryptic peptides were separated by nanoscale C18 reverse chromatography coupled on line to an Orbitrap Fusion Tribrid mass spectrometer (Thermo Scientific) via a nanoelectrospray ion source (Nanospray Flex Ion Source, Thermo Scientific). Peptides were loaded on a 20 cm 75–360 μm inner-outer diameter-fused silica emitter (New Objected) packed in-house with ReproSil-Pur C18-AQ, 1.9 μm resin (Dr Maisch GmbH). The column was installed on a Dionex Ultimate3000 RSLC nanoSystem (Thermo Scientific) using a MicroTee union formatted for 360 μm outer diameter columns (IDEX) and a liquid junction. The spray voltage was set to 2.15 kV. Buffer A was composed of 0.5% acetic acid and buffer B of 0.5% acetic acid, 80% acetonitrile. Peptides were loaded for 17 min at 300 nl/min at 5% buffer B, equilibrated for 5 min at 5% buffer B (17–22 min) and eluted by increasing buffer B from 5 to 15% (22–87 min) and 15–38% (87–147 min), followed by a 10 min wash to 90% and a 5 min regeneration to 5%. Survey scans of peptide precursors from 400 to 1500 m/z were performed at 120 K resolution (at 200 m/z) with a 1.5 × 10^5^ ion count target. Tandem mass spectrometry was performed by isolation with the quadrupole with isolation window 1.6, HCD fragmentation with normalized collision energy of 30, and rapid scan mass spectrometry analysis in the ion trap. The MS^2^ ion count target was set to 10^4^ and the max injection time was 35 ms. Only those precursors with charge state 2–7 were sampled for MS^2^. The dynamic exclusion duration was set to 60 s with a 10 ppm tolerance around the selected precursor and its isotopes. Monoisotopic precursor selection was turned on. The instrument was run in top speed mode with 3 s cycles. All data were acquired with Xcalibur software.

### Mass spectrometry data processing and analysis

RAW mass spectrometry files were processed as previously described^22^ with the MaxQuant computational platform^72^ (version 1.5.2.8) using label-free quantitation (LFQ). Peptides were identified using the Andromeda search engine by querying the human UniProt database with a 1% false discovery rate (FDR) cutoff both at peptide and protein level. Potential contaminants and reverse hits were removed using Perseus (version 1.6.12). LFQ values were log_2_-transformed and the biological replicates were grouped based on cell type and treatment. For analysis of unstimulated samples, proteins with a minimum of 3 valid values in at least one cell type were included for further analysis (Supplementary Table 1). For analysis of samples with or without CD3/CD28-mediated restimulation, a minimum of 3 valid values in at least one sample group was required to include proteins for further analysis (Supplementary Table 1). Missing value imputation was performed separately for each sample, replacing missing values by random numbers drawn from the lower part of the normal distribution (width = 0.3, shift = 1.8). The proteomics experiment was conducted with three biological replicates per sample group, but one sample, i.e. restimulated iTreg cells from donor 1, was excluded from the analysis due to lower protein content that led to fewer detected proteins. PCA revealed that this sample was an outlier.

Proteomics data were analyzed using Qlucore Omics Explorer (version 3.8) to perform PCA and ANOVA test followed by a Benjamini–Hochberg multiple testing correction with a 5% FDR. After z-score normalization, clusters of proteins with similar expression patterns were identified using hierarchical clustering and visualized in heat maps. Protein clusters were subjected to overrepresentation analysis to assess enrichment of GO biological processes using the STRING app (version 1.6.0) in Cytoscape (version 3.9.0). To refine the analysis of biological processes, a redundancy cutoff was applied when significant GO terms contained large overlap based on enriched genes. STRING was used to visualize protein networks. The proteomic response to restimulation was analyzed for each cell type using GO biological processes in GSEA software (version 4.1.0). GSEA was performed using default parameters and gene set permutations. To identify related groups of biological processes that were differentially regulated following restimulation of T cells, the EnrichmentMap app (version 3.3.2) was used in Cytoscape. The largest gene set clusters were displayed using manually assigned annotations to summarize the biological processes represented by the clusters.

### Statistical analysis

Data were analyzed using GraphPad Prism (version 9.0.1), except for proteomics data. Statistical analyses were performed as indicated in the figure legends. Log_2_ transformation was performed when data were not normally distributed. Data are presented as mean ± SEM and a two-sided *p* < 0.05 was considered statistically significant.

## Supplementary Information

Supplementary Figures.

Supplementary Table 1.

Supplementary Table 2.

Supplementary Table 3.

Supplementary Table 4.

## Data Availability

Proteomics data on tTreg and Tconv cells have been deposited to the ProteomeXchange Consortium (http://proteomecentral.proteomexchange.org) via the PRIDE repository^73^ with the dataset identifier PXD007745, as outlined before^22^. Proteomics data on iTreg cells are also available via ProteomeXchange with the dataset identifier PXD038302.
