# Supplementary material for: Proteomics reveals unique identities of human TGF-β-induced and thymus-derived CD4+ regulatory T cells
Source: Sci Rep. 2022 Nov 24;12:20268. doi: 10.1038/s41598-022-23515-z (PMC9700829; doi:10.1038/s41598-022-23515-z)
Supplement: Supplementary file 1 — Supplementary Figures. [file 41598_2022_23515_MOESM1_ESM.pdf]

Supplementary Information

Proteomics reveals unique identities of human TGF-β-induced and thymus-derived CD4+ regulatory T cells

Mark Mensink[1], Ellen Schrama[1], Eloy Cuadrado[2],

Derk Amsen[2], Sander de Kivit[1,†,*] and Jannie Borst[1,†,*]

[1]Department of Immunology and Oncode Institute, Leiden University Medical Center, Leiden, The Netherlands

Department of Hematopoiesis[2], Sanquin Research and Landsteiner Laboratory, Amsterdam UMC, University of Amsterdam, Amsterdam, The Netherlands

[†]These authors share senior authorship

*** Correspondence:** Dr Sander de Kivit (S.de_Kivit@lumc.nl) or Prof Jannie Borst (J.G.Borst@lumc.nl)

Supplementary Figure 1

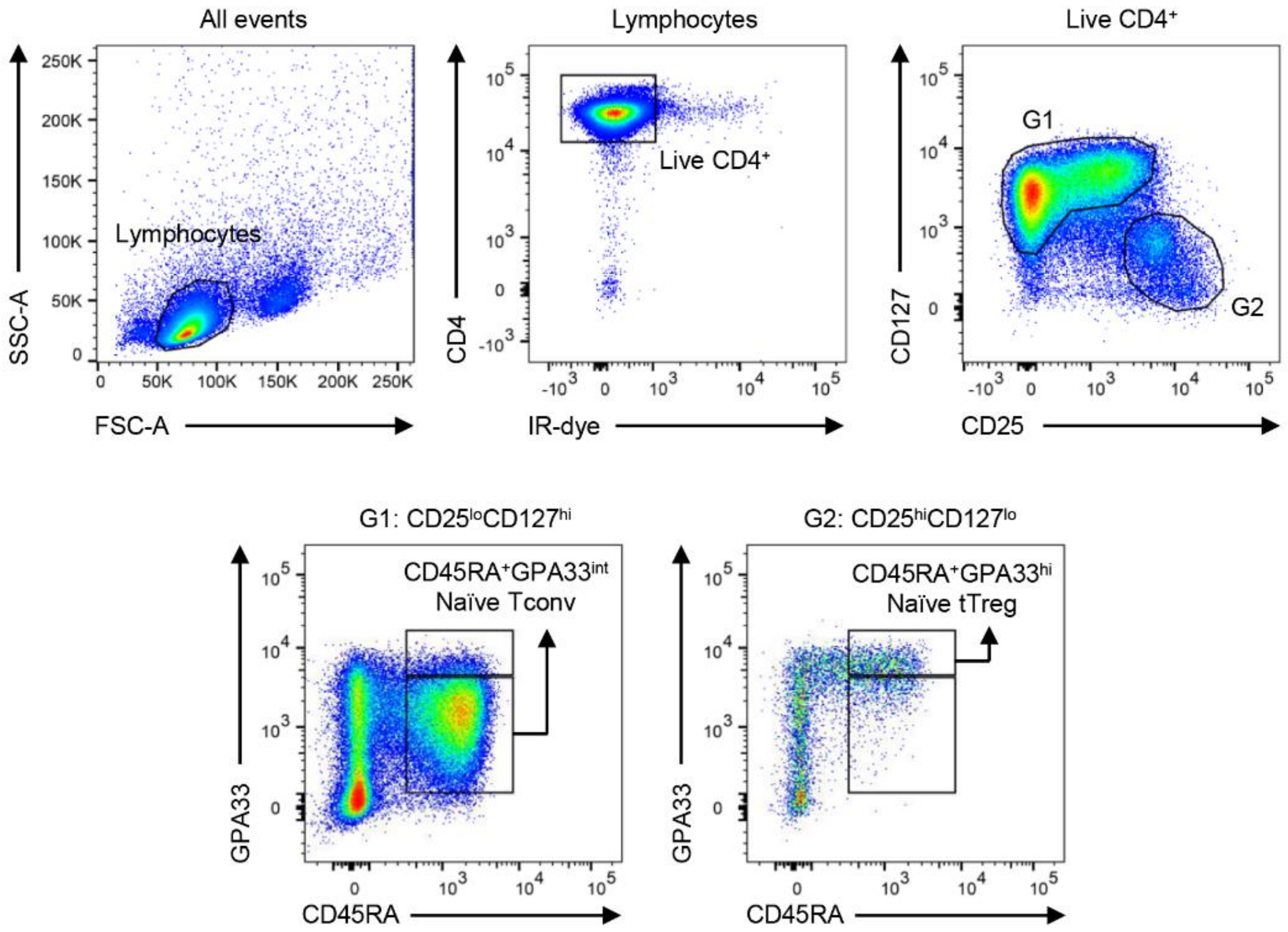

Supplementary Figure 1. Gating strategy to sort naïve Tconv and naïve tTreg cells from human peripheral blood by flow cytometry. Pre-enriched total $CD4^{+}$ T cells from human peripheral blood were first separated into live $CD4^{+}CD25^{lo}CD127^{hi}$ cells within gate 1 (G1) and live $CD4^{+}CD25^{hi}CD127^{lo}$ cells within gate 2 (G2). Subsequently, $CD45RA^{+}GPA33^{int}$ naïve Tconv cells and $CD45RA^{+}GPA33^{hi}$ naïve tTreg cells were selected within G1 and G2, respectively.

Supplementary Figure 2

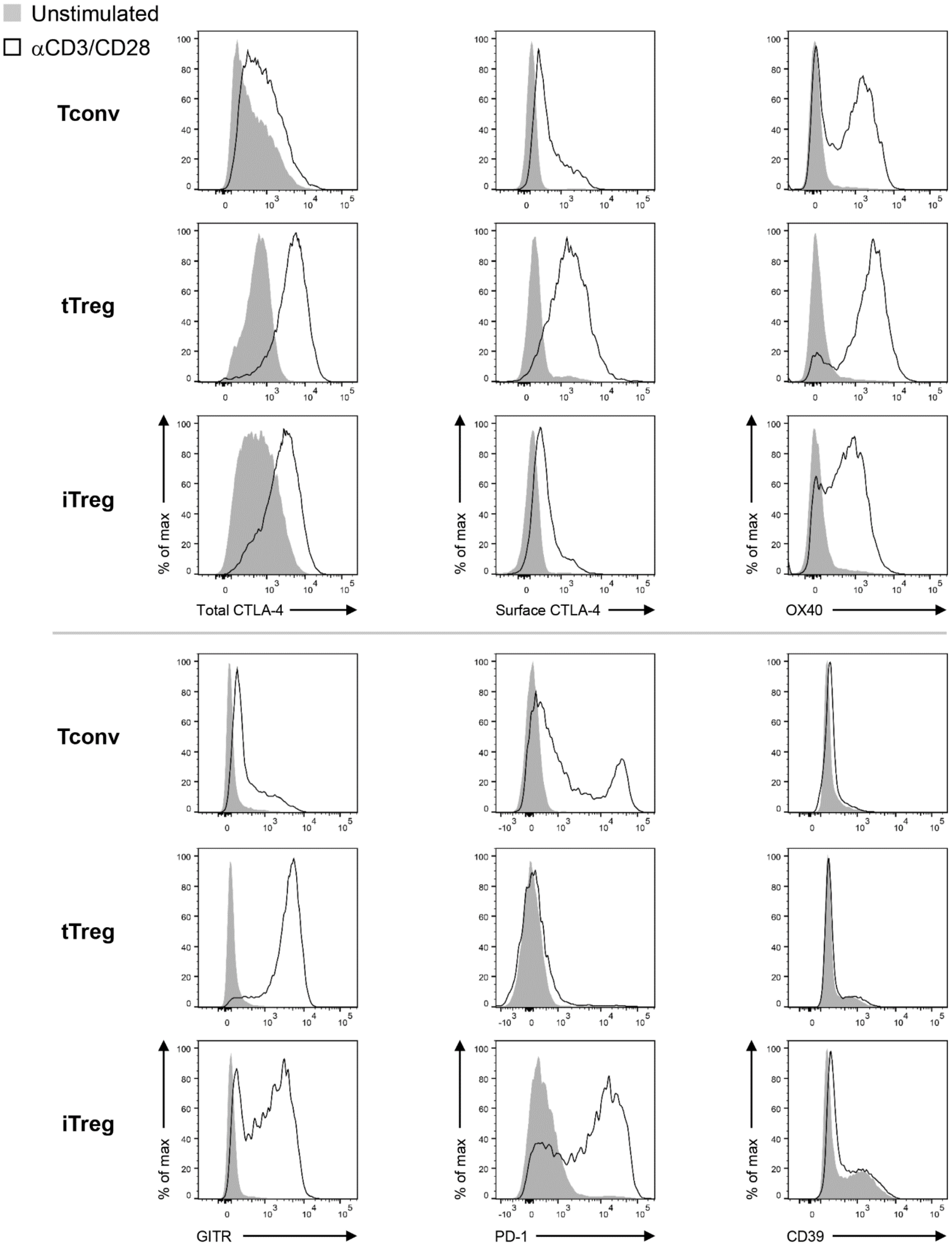

Supplementary Figure 2. Representative flow cytometry histograms showing protein expression of total CTLA-4 (n = 13), surface CTLA-4 (n = 8), surface OX40 (n = 8), surface GITR (n = 8), surface PD-1 (n = 8) and surface CD39 (n = 8) on Tconv, tTreg and iTreg cells that were restimulated via CD3/CD28 or not.

Supplementary Figure 3

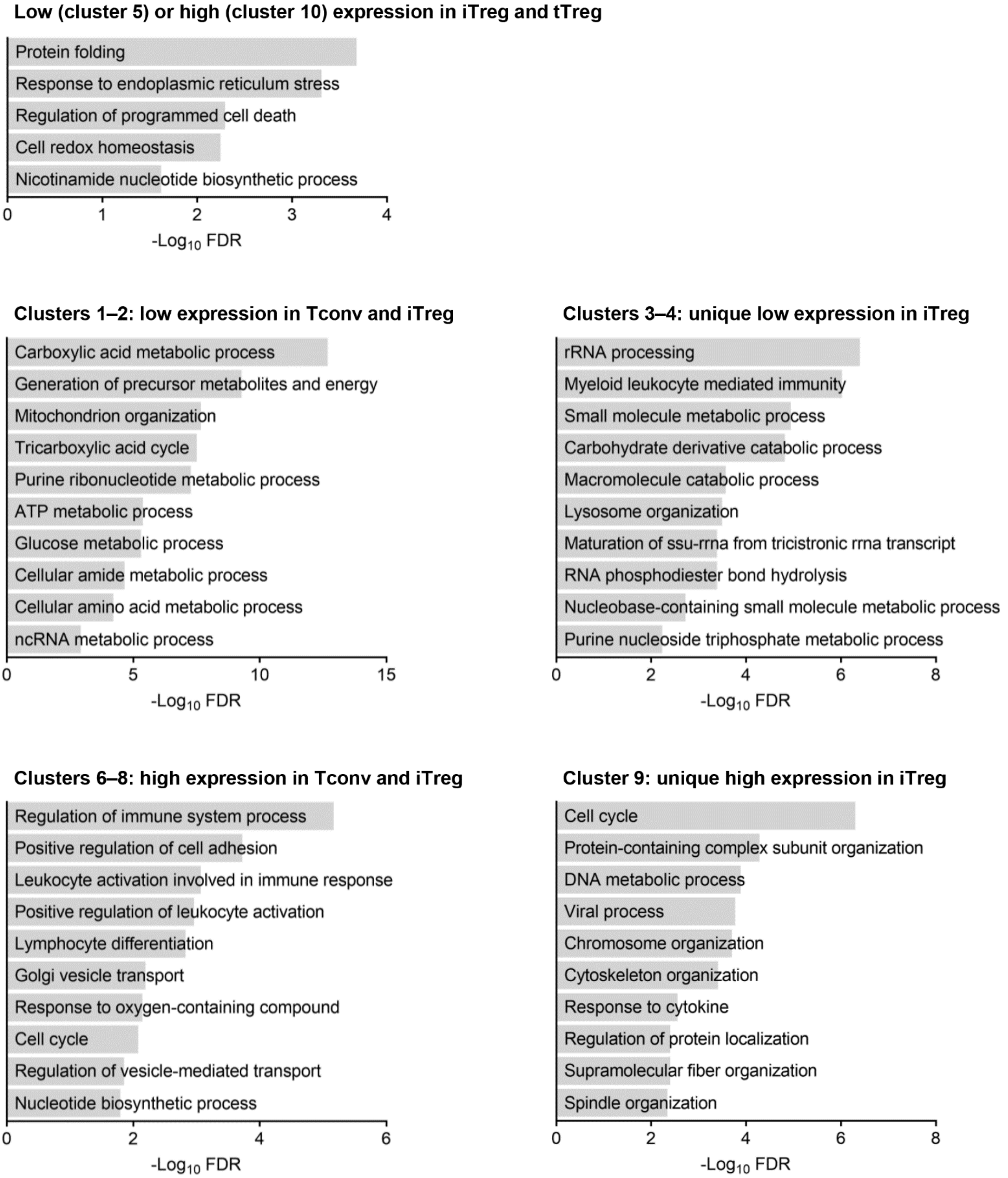

Supplementary Figure 3. GO biological processes enrichment analyses of indicated clusters of proteins from **Figure 3b** that were differentially expressed between unstimulated Tconv, tTreg and iTreg cells (FDR < 0.05).

Supplementary Figure 4

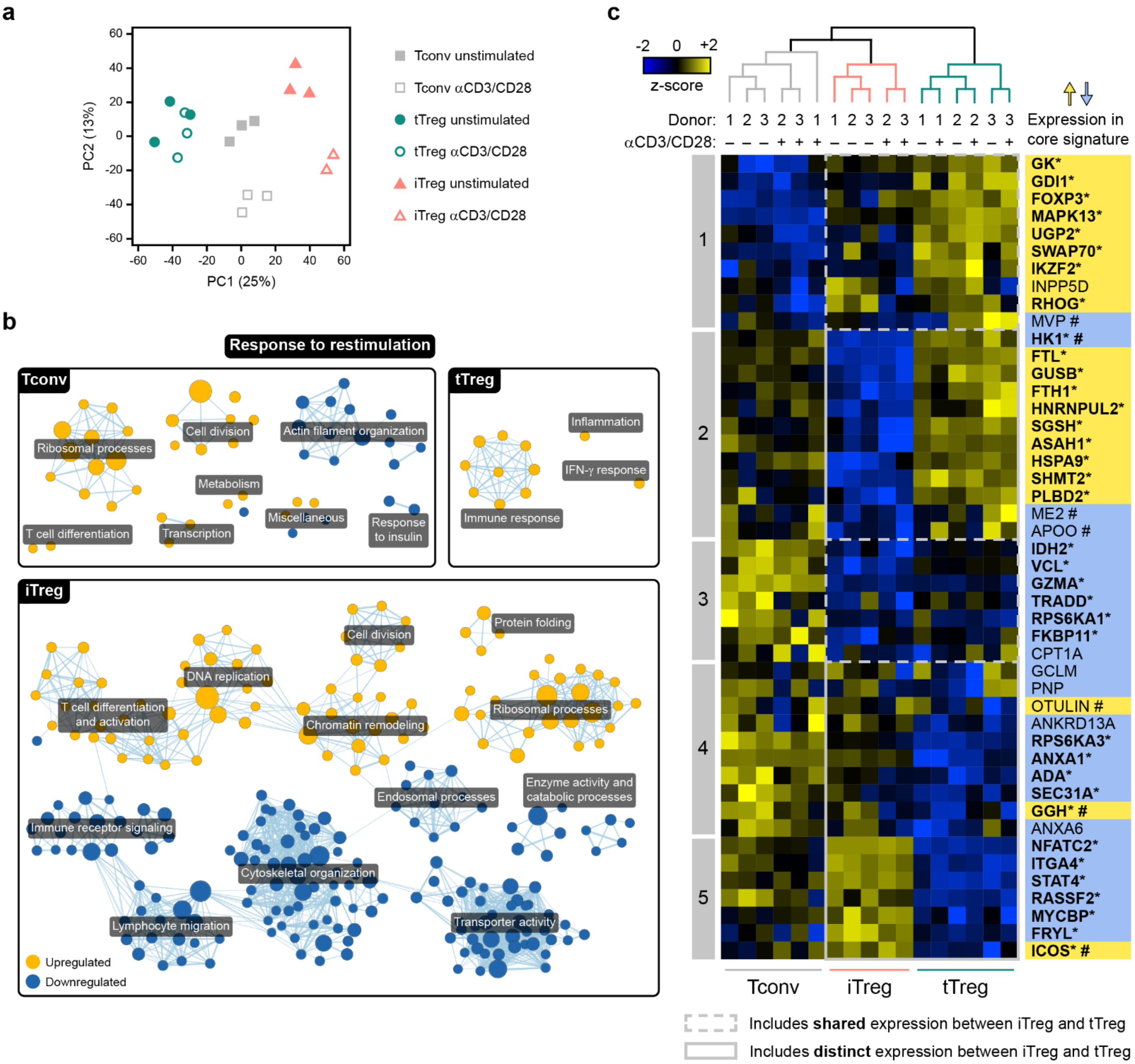

Supplementary Figure 4. Analysis of the proteomic responses of Tconv, tTreg and iTreg cells to CD3/CD28-mediated restimulation. **(a)** PCA plot of the proteomes of indicated cell populations that were restimulated or not (*n* = 3, *n* = 2 only for CD3/CD28-restimulated iTreg cells). **(b)** GSEA enrichment maps of the proteomic responses of Tconv, tTreg or iTreg cells to CD3/CD28-mediated restimulation, showing enriched GO biological processes in yellow (upregulated) and blue (downregulated). Gene sets are depicted as nodes, of which the size represents the number of genes in the gene set. Gene sets sharing a large number of genes are clustered and are connected by lines of which the thickness corresponds with the number of shared genes. Clusters of similar biological processes are annotated using summarizing terms. **(c)** Heat map showing hierarchical clustering of 46 detected proteins of the Treg cell core proteomic signature[22] in Tconv, tTreg and iTreg cells that were CD3/CD28-stimulated or not. Samples are colored according to cell type as in **a**. Z-scores showing relative expression values are color-coded. Numbered boxes (1–5) indicate clusters of proteins. For reference, protein names are colored according to high (yellow) or low (blue) relative expression in the Treg cell core signature as shown in **Figure 4a**. Significantly differential expression (FDR < 0.05) is indicated by asterisks and bold letters. Proteins are marked (#) when expression in *in vitro*-expanded tTreg cells from the present dataset deviates from the core signature derived from *ex vivo* Treg cells[22].

Supplementary Figure 5

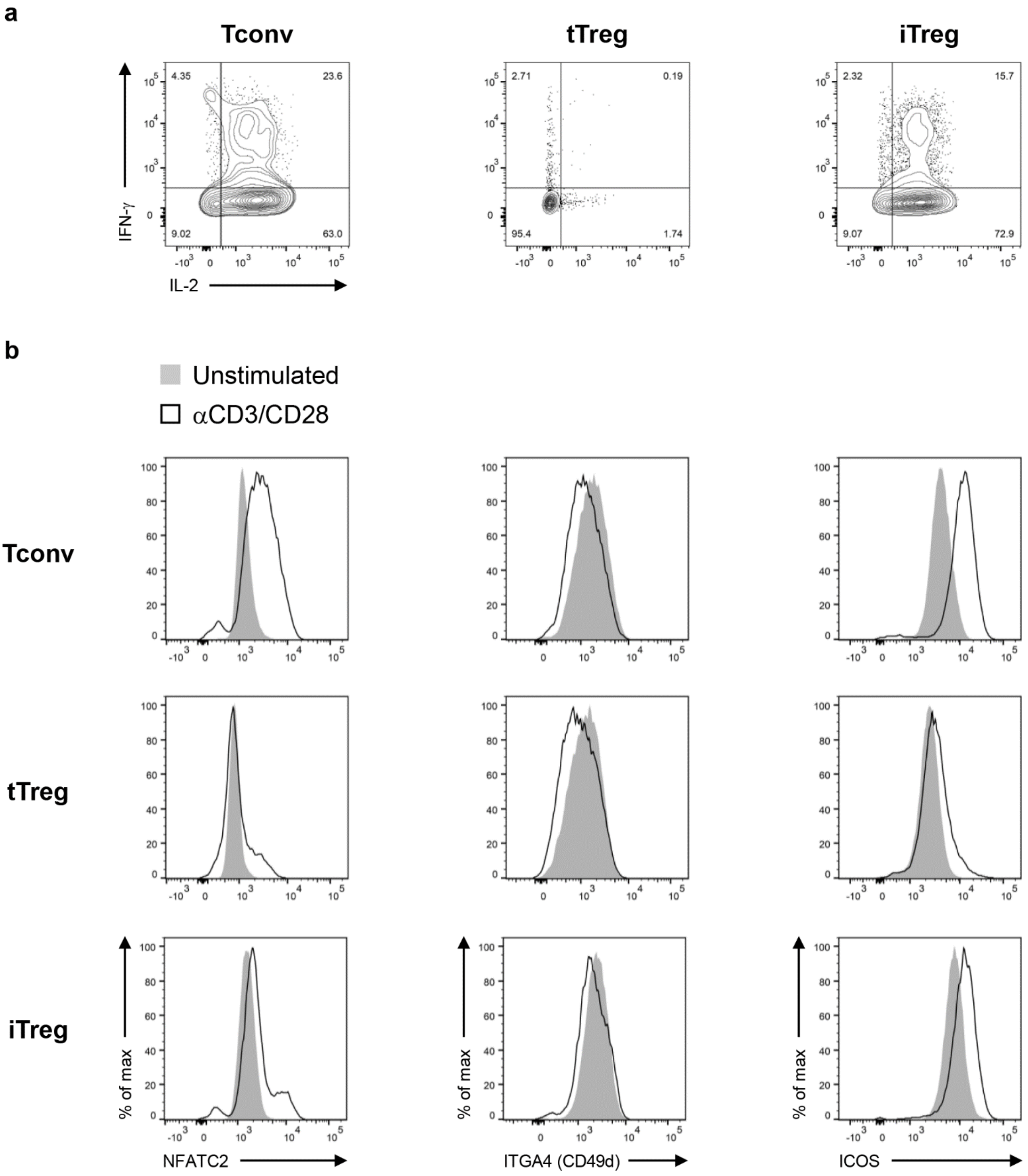

Supplementary Figure 5. (a) Representative flow cytometry contour plots showing intracellular protein expression of IL-2 and IFN-γ in PMA/ionomycin-treated Tconv, tTreg and iTreg cells (*n* = 4). Gates are set based on samples that were not treated with PMA/ionomycin and quadrants depict percentages. **(b)** Representative flow cytometry histograms showing protein expression of total NFATC2 (*n* = 4), surface ITGA4 (CD49d) (*n* = 5) and surface ICOS (*n* = 5) on Tconv, tTreg and iTreg cells that were restimulated via CD3/CD28 or not for 24 h.

Supplementary Figure 6

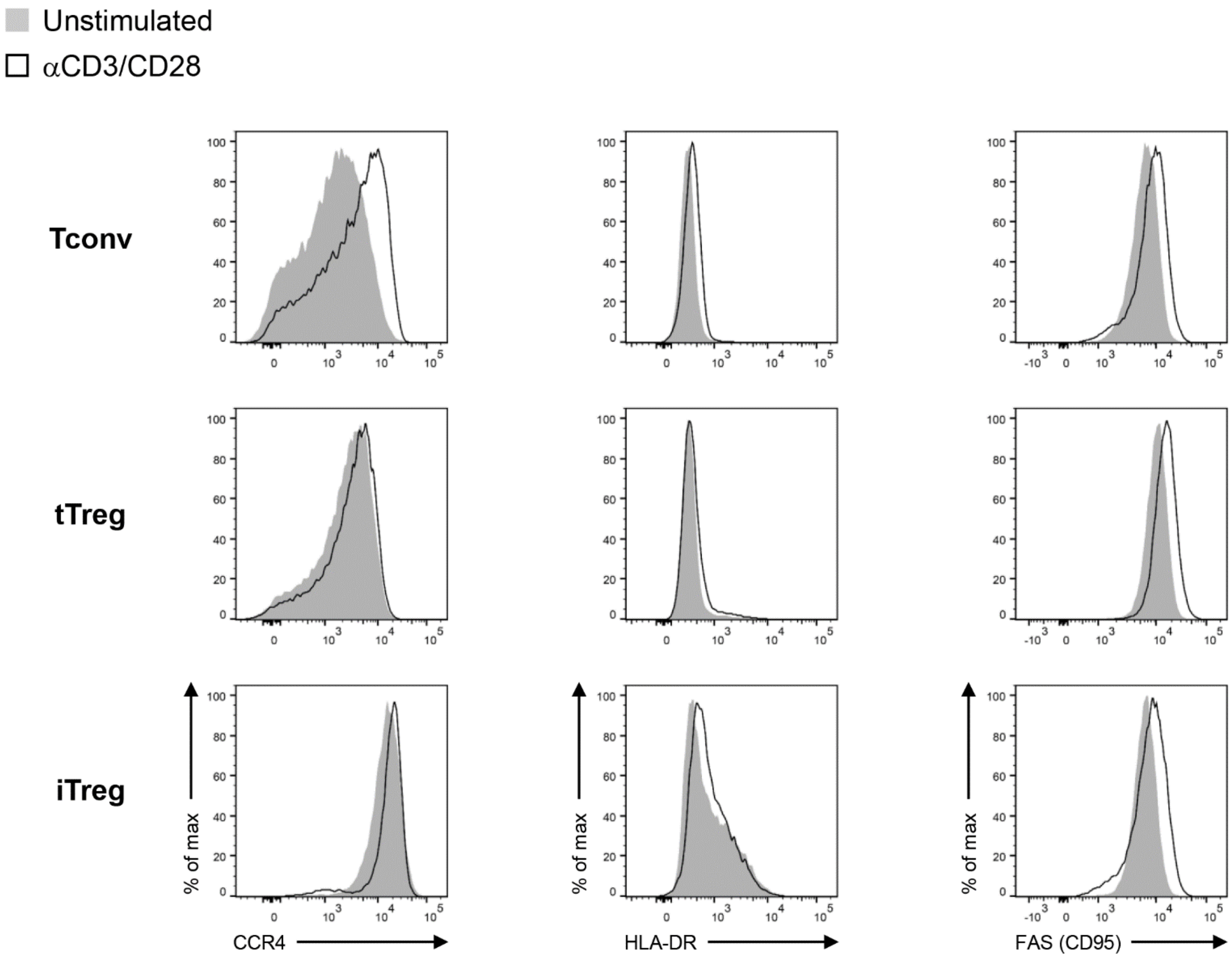

Supplementary Figure 6. Representative flow cytometry histograms showing protein expression of surface CCR4 (n = 4), surface HLA-DR (n = 4) and surface FAS (CD95) (n = 4) on Tconv, tTreg and iTreg cells that were restimulated via CD3/CD28 or not for 24 h.

Supplementary Figure 7

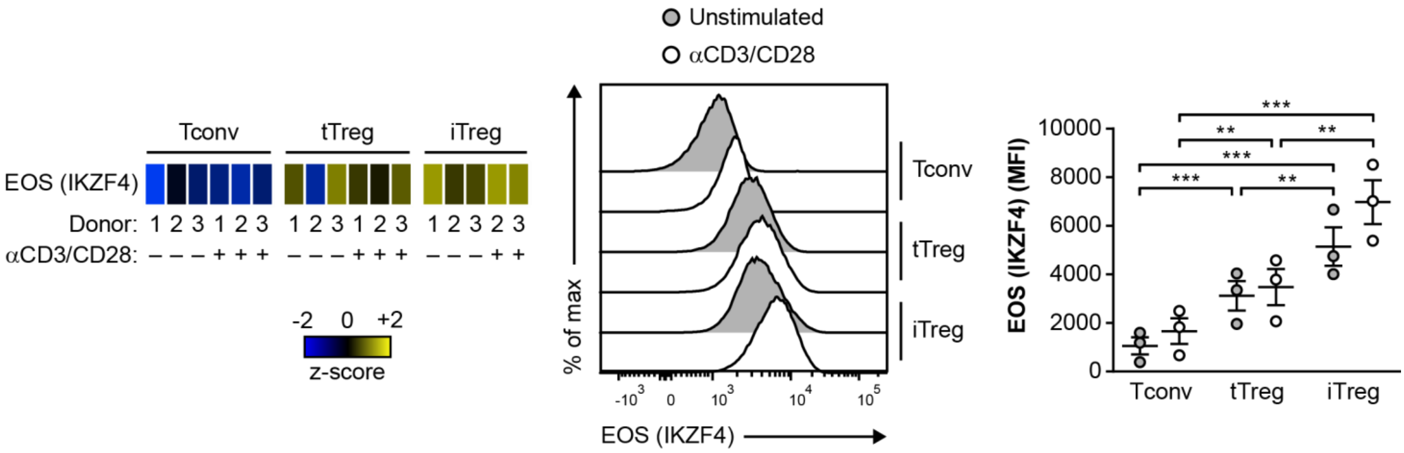

Supplementary Figure 7. EOS (IKZF4) expression in indicated cell populations that were restimulated via CD3/CD28 or not for 24 h, as measured by proteomics (left panel) and flow cytometry, showing representative histograms (middle panel) and quantification (right panel) (n = 3). Right panel: MFI is depicted on the y-axis. Two-way ANOVA with Tukey's *post hoc* test was used for statistical analysis. Data are presented as mean ± SEM. Sample size (n) represents cells from individual donors, analyzed in independent experiments. **$p < 0.01$, ***$p < 0.001$.
